# Influence of training in the use and generation of evidence on episiotomy practice and perineal trauma

**DOI:** 10.1016/j.ijgo.2010.04.035

**Published:** 2010-10

**Authors:** Jacqueline J. Ho, Porjai Pattanittum, Robert P. Japaraj, Tari Turner, Ussanee Swadpanich, Caroline A. Crowther, for the SEA-ORCHID Study Group

**Affiliations:** a Department of Paediatrics, Penang Medical College, Penang, Malaysia; b Department of Biostatistics and Demography, Khon Kaen University, Khon Kaen, Thailand; c Hospital Raja Permaisuri Bainun, Ipoh, Malaysia; d Monash Institute of Health Services Research, Monash University, Melbourne, Australia; e Department of Obstetrics and Gynecology, Khon Kaen Hospital, Khon Kaen, Thailand; f Discipline of Obstetrics and Gynaecology, University of Adelaide, Adelaide, Australia

**Keywords:** Capacity building, Episiotomy, Low-income countries, Practice change

## Abstract

**Objective:**

To examine episiotomy practices before and after a multi-component intervention designed to support the use and generation of research evidence in maternal and neonatal health care.

**Methods:**

Set in 9 centers across 4 Southeast Asian countries, a retrospective survey was performed for 12 recommended pregnancy/childbirth practices and 13 outcomes of women in each center before and after intervention. Qualitative interviews were conducted to assess staff awareness and experience in evidence-based practice.

**Results:**

There were significant decreases in the rate of episiotomy, from 64.1% to 60.1% (risk difference [RD] –4.0; 95% confidence interval [CI], –5.8 to –2.2) for all women and from 92.2% to 80.7% (RD –11.5; 95% CI, –13.4 to –9.6) for nulliparous women. Severe trauma decreased from 3.9% to 1.9% (RD –2.0; 95% CI, –2.7 to –1.4) for all women and from 6.7% to 3.0% (RD –3.7; 95% CI, –4.9 to –2.5) for nulliparous women. The frequency of intact perineum increased from 12.4% to 15.6% (RD 3.2; 95% CI, 1.9–4.6) for all women and from 1.7% to 8.0% (RD 6.3; 95% CI, 5.0–7.5) for nulliparous women.

**Conclusion:**

An intervention based on understanding and using the best available evidence can result in significant improvements in care and health outcomes.

## 1 Introduction

Despite being a form of trauma, episiotomy is one of the most common surgical procedures performed on women [1]. High episiotomy rates, some close to 100%, have been reported in North America and Asia [2,3].

Three Cochrane Systematic Reviews provide evidence regarding the practice of episiotomy—comparing restrictive and routine episiotomy [4], suturing techniques [5], and suturing materials [6]. Restrictive episiotomy reduces by approximately 30% severe perineal trauma, need for suturing, and healing complications, without any increase in pain, dyspareunia, or urinary incontinence—despite an almost doubling of the risk of anterior perineal trauma. The benefit of a restrictive practice outweighs the potential harm [4]. If perineal repair is required, a continuous suture results in less pain than does interrupted suturing, although the technique is more difficult [5]; furthermore, the use of synthetic absorbable suture material causes less pain but is more likely to require removal [6].

According to Pakenham-Walsh et al. [7], “providing access to reliable health information for workers in developing countries is potentially the single most cost-effective and achievable strategy for sustainable improvement in health care.” Turning evidence into practice is a complex process; strategies found to be effective include the use of local opinion leaders [8], educational outreach [9], and audit and feedback [10].

The aim of the present study was to examine episiotomy practices used for women undergoing normal vaginal delivery at term in Southeast Asia before and after a multi-component intervention designed to support the use and generation of research evidence in maternal and neonatal health care.

## 2 Materials and methods

The study was part of the South East Asia Optimising Reproductive and Child Health in Developing Countries (SEA-ORCHID; http://www.seaorchid.org) project. The SEA-ORCHID project was designed to answer the question “can the health of mothers and babies in Thailand, Indonesia, Philippines, and Malaysia be improved by increasing capacity for the synthesis of research, implementation of effective interventions, and identification of gaps in knowledge needing further research in those countries?”

The present study was undertaken in 9 centers (Khon Kaen University, Khon Kaen Hospital, and Kalasin Hospital in Thailand; Universiti Sains Malaysia and Hospital Raja Permaisuri Bainun Ipoh in Malaysia; Philippines General Hospital and Jose Fabella Hospital in the Philippines; and Dr Sardjito Hospital and Sleman District Hospital in Indonesia) across 4 Southeast Asian countries, with support from 3 Australian Universities (University of Sydney, University of Adelaide, and Monash University). There were 3 main phases to the SEA-ORCHID project, the details and methods for which have been described previously [11]. Briefly, in the first (pre-intervention) phase, primary data were obtained via an audit of 12 areas of recommended practice (including restrictive episiotomy) and 13 health outcomes in pregnancy, childbirth, and neonatal care (including intact perineum). Secondary data were obtained by examining staff knowledge of evidence-based practice, access to evidence, research activity, guideline development, systematic review activity, and undergraduate teaching in evidence-based practice. The second phase comprised an educational intervention, and in the third phase the audit was repeated.

The intervention was tailored to meet the specific needs of each site. It focused on evidence users (clinicians and policy makers), generators (systematic review, guideline development, and improving research infrastructure), and educators (teachers and trainers).

Two or 3 clinical educators from each country, usually mid-career and selected for their ability to act as opinion leaders, underwent a period of training in Australia and then carried out site-specific activities to support building capacity in evidence-based practice tailored to perceived need. Training included interactive evidence-based practice workshops, and some centers used poster displays of evidence, periodic audit, and feedback of rates. All categories of staff were involved. In addition, training was provided on systematic reviewing, guideline development, and input into undergraduate curricula. The academic exchanges consisted of 1–2-month fellowships to the Australian centers for 23 Southeast Asian participants, and teaching tours provided by Australian educators and investigators. The centers did not include specific training on episiotomy, and direct orders to stop or reduce the frequency with which this procedure was performed were not given. Some centers carried out specific training on protecting the perineum without episiotomy, in addition to continuous suturing technique.

The primary audit of 1000 deliveries in each center recorded whether episiotomy was performed, the type of suture material and suture technique used for repair, and the degree of perineal trauma. All women who had a normal vaginal delivery at term were included. Data were extracted from medical records by trained data collectors using a specially designed format, then manually entered into a secure web-based database. Pre-intervention data were collected between April 1 and October 31, 2005, and post-intervention data were obtained between January 1 and June 30, 2008.

Perineal trauma was defined as trauma of the perineum during childbirth that required stitches. Vaginal and perineal tears were classified as follows: first-degree tears involved the vaginal mucosa and connective tissue; second-degree tears included the underlying muscles; third-degree tears involved complete transaction of the anal sphincter (confirmed by clinical observation/examination); and fourth-degree tears involved the rectal mucosa. Third- or fourth-degree trauma was classified as severe. Episiotomy was defined as surgical enlargement of the vagina via perineal incision during delivery. The technique of skin closure was classified as either continuous (simple, non-locking, continuous subcuticular sutures) or interrupted (interrupted transcutaneous sutures).

Qualitative data were collected via in-depth interviews conducted during the final 9 months of the intervention (from July 1, 2007, to February 28, 2008). The interviews were based on a pre-specified protocol and were designed to explore the interviewees’ awareness of and experience in undertaking evidence-based practice, carrying out research, and developing guidelines. Two members of the Australian team with experience in qualitative methods and practice change conducted semi-structured face-to-face interviews with maternity and neonatal clinicians at each participating hospital. At each hospital, 2 junior and 2 senior doctors, and 2 junior and 2 senior nurses/midwives were interviewed. In Indonesia and Thailand, interpreters were used during the interviews.

All interviews were audio recorded, transcribed, and anonymized; data were analyzed in emerging themes using NVivo (QSR International, Melbourne, Australia).

Rates were given as percentages, and the difference pre- and post-intervention was described as a risk difference (RD) and a 95% confidence interval (CI) of the RD. This was done for all women and for nulliparous women. Data for each country were pooled and the RD for intact perineum was adjusted for maternal age and parity. *P* < 0.05 was considered to be statistically significant.

The SEA-ORCHID project was approved by the ethics committee at the project administration center, University of Sydney, and at each participating center. Informed written consent was obtained before the interviews were conducted.

## 3 Results

Data were available for 11 016 women who underwent vaginal delivery at term. The baseline characteristics of women and infants were similar pre- and post-intervention, except for those of nulliparous women in both of the Malaysian centers (Table 1).

Five centers reported a significant decrease in the rate of episiotomy post-intervention, 2 reported no change, and 2 reported a significant increase. The initial rate of episiotomy across all centers was 64.1%, and this decreased to 60.1% post-intervention (RD –4.0; 95% CI, –5.8 to –2.2). For nulliparous women, there was a decrease in 6 centers, with the overall rate decreasing from 92.2% to 80.7% (RD –11.5; 95% CI, –13.4 to –9.6) (Table 2).

There was a significant increase in the rate of intact perineum in 4 centers, a trend toward significance in 2 centers, and an decrease in 1 centre—resulting in an overall increase from 12.4% to 15.6% (RD 3.2; 95% CI, 1.9–4.6) for all women and from 1.7% to 8.0% (RD 6.3; 95% CI, 5.0–7.5) for nulliparous women. In 1 center, there was a significant decrease in the overall rate of intact perineum, but an increase among nulliparous women (Table 2). When data were pooled for each country, Thailand and the Philippines showed an improvement in the rate of intact perineum; however, the improvement remained significant only for the Philippines after adjustment for parity and maternal age (Table 3).

The pre-intervention rate of severe trauma ranged from 0.0% to 36.9% across the 9 centers. Overall, the rate decreased from 3.9% to 1.9% (RD –2.0; 95% CI, –2.7 to –1.4) for all women. This reduction was greatest for nulliparous women: 6.7%–3.0% (RD –3.7; –4.9 to –2.5). The rate of severe trauma also decreased for women with and women without episiotomy, with a greater reduction in the latter group (Table 4).

The rate of use of continuous sutures was more than 90% both pre- and post-intervention in 4 centers. Three centers reported a significant decrease in the use of continuous suture, and only 1 experienced a significant increase. The remaining center reported a non-significant increase. Overall, the use of continuous sutures decreased from 71.5% to 63.6% (Table 5).

Chromic catgut was the most common suture material used for perineal repair. Polyglycolic acid was used in 60 (1.2%) of the 5070 pre-intervention births and 429 (9.8%) of the 4358 post-intervention births (Table 5). A change to polyglycolic acid was implemented in 6 centers, 2 of which achieved a significant increase.

In total, 75 individual, 25 pair, and 11 group interviews were conducted. Episiotomy was mentioned in 50 interviews, distributed equally across countries. In some hospitals, clinicians reported that practice regarding episiotomy had changed substantially as a result of the project: “Like the episiotomy. I think last time we did routinely do it but, after the introduction of the SEA-ORCHID, now we selectively practice this.”

Several clinicians in other hospitals indicated that they were aware that there was evidence that episiotomy should be undertaken selectively rather than routinely, but that it was still being carried out routinely in their hospitals. One frequently reported reason for routine episiotomy was fear of tearing. Some nurses and junior doctors were concerned about the response of senior doctors if significant tearing occurred when episiotomy was not performed. Episiotomies were preferred to tears because they were easier to repair: “Maybe they are not [relying] on the evidence, because sometimes when we give birth without episiotomy the wound, the vagina, is teared [sic] and it's difficult to repair; that is a bad experience for them.” There was also an increase in difficulties associated with suturing tears following other practice changes such as reducing perineal shaving.

The need for preparation of the perineum (e.g. through perineal massage) was mentioned frequently, and it was suggested by some clinicians that research into selective episiotomy was applicable to other populations in which women underwent appropriate perineal preparation. The applicability of research into episiotomy in different populations was also questioned for other reasons: “For example, episiotomy because the size of the vagina is different between [local women] and foreigners.”

The need to train junior staff in episiotomy technique was another reported barrier to change in several hospitals: “This hospital is a referral and teaching hospital, so the residents study here…so the episiotomy, they need to do it so [they] know by themselves how to stitch the patients. That's why it is difficult to eradicate episiotomy.”

Several interviewees noted that a lack of time led some clinicians to perform episiotomies unnecessarily: “Sometimes they are impatient. Because you must have patience to wait for patients to deliver normally vaginally, she needs to take time. They want to fasten [sic] the delivery.”

In several hospitals, another barrier to change was the use of outdated protocols still recommending routine episiotomy. Three approaches were suggested as being particularly helpful in terms of changing practice regarding episiotomy. In 2 hospitals at which clinicians believed there had been a successful change in practice, staff had undertaken research projects to investigate the impact of moving from routine to selective episiotomy at the hospital. This was felt to be important in demonstrating that the results of research conducted elsewhere also applied locally.

Changes in financial incentive were also noted as being effective at modifying episiotomy practice: “[Before] if you put…‘vaginal delivery’, that may not be compensated. But, if you put maybe ‘episiotomy and repair,’ it will be compensated. So now they don't compensate [episiotomy] anymore…you can only provide normal delivery and you will be compensated.”

Finally, several clinicians mentioned the value of visits to other hospitals, undertaken by themselves or colleagues, to witness first-hand that selective episiotomy was safe.

## 4 Discussion

In the present study, a multi-component intervention centered around understanding and using the best available evidence resulted in significant improvements in restrictive episiotomy practice and an increase in the rate of intact perineum in both parous and nulliparous women undergoing vaginal delivery at term.

Before the intervention, episiotomy rates were high in 8 of the 9 centers, and across all centers almost all nulliparous women underwent episiotomy. Following the intervention, although significant reductions were achieved, rates were still high. In a survey of Canadian hospitals practicing restricted episiotomy, the median rates reported were 62% for nulliparous and 40% for multiparous women [12]. In 1989, 39% of women undergoing vaginal delivery in the USA underwent episiotomy [1], compared with 28%–83% in the present study. It was gratifying that all centers reporting a decrease in the rate of episiotomy experienced an increase in the rate of intact perineum, which was associated with an overall reduction in the rate of severe trauma among both the women who underwent episiotomy and those who did not. Although the 2.9% increase in the rate of intact perineum (9.2% for nulliparous women) corresponded to thousands of women in Southeast Asia experiencing childbirth without the pain and complications of episiotomy, there are still many more women who could benefit from this intervention. Similarly, although this improvement translated into less use of resources and health practitioner's time, further reductions are possible.

The fact that each center designed and implemented their intervention could explain the wide variation in RD pre- and post-intervention. The intervention focused on the skills needed to understand and implement evidence, resulting in a varied emphasis on specific practices depending on perceived needs. This could also be the reason why some centers reported no decrease or even an increase in episiotomy rate. In addition, it might explain why the overall decrease in episiotomy rate (from 64.1% to 60.1%) was modest. However, the advantage of this approach is that centers now have the skills to identify and implement new evidence. An unexpected benefit was that episiotomy rate was included as a national indicator in the Quality Improvement Programme of Malaysia. Thus, Malaysia could expect a decrease in episiotomy rate nationwide.

The intervention period was 2.5 years, and some changes may take longer to implement: for example, changes to midwifery and undergraduate medical curricula/subsequent graduation of students taught the new curricula. Furthermore, teachers of restrictive episiotomy practice also require initial training on conducting a normal birth without perineal trauma. This was evidenced by the fact that data collection at 1 of the centers at which there was an increase in episiotomy rate coincided with the entry of new residents trained in routine episiotomy. In addition to differences in intervention, differences in the enthusiasm of key staff could contribute to the wide variation seen between centers.

Several centers attempted to change from catgut to synthetic suture material, but the associated cost prohibited implementation in most centers. In 1 country, it was discussed at national level and a decision was made that it was too expensive to implement. In 2 countries, patients had to pay for the suture material themselves; despite this, 2 centers reported a significant change (data not shown).

The interviews identified a range of barriers to restrictive episiotomy practice. These included fear of tearing, difficulty in repairing tears compared with cuts, lack of perineal preparation, applicability of research carried out in a different population, lack of time, and need to train junior staff. Fear of tearing and difficulty associated with repair were reasons for episiotomy originally being introduced. The applicability of evidence between different populations is a valid concern, and it is reasonable to query whether there could be anatomic differences in perineal structure among Southeast Asian women that might affect applicability. Higher rates of episiotomy and perineal trauma have been reported for Asian women than for non-Asian women [13–16]. One center had successfully implemented restricted episiotomy practice pre-intervention. During the study, most centers were able to implement a restrictive practice without an increase in severe trauma and, in fact, many experienced a decrease. The results show that evidence for restricted episiotomy can be applied beneficially to Southeast Asian populations.

In conclusion, an intervention to increase the use and generation of evidence can result in significant improvements in episiotomy practice and a reduction in the rate of perineal trauma among women. The model could be adapted to improve the uptake of evidence-based practice in other centers and in other disciplines.

## Conflict of interest

The authors have no conflicts of interest.

## Figures and Tables

**Table 1:** Characteristics of women and infants for all full-term normal births in pre- and post-intervention surveys.a,b

	Thailand				Philippines			Malaysia			Indonesia		
	Center 1	Center 2	Center 3	All	Center 1	Center 2	All	Center 1	Center 2	All	Center 1	Center 2	All
Mother													
Pre-intervention	919 (55.0)	950 (56.4)	956 (55.1)	2825 (55.5)	947 (86.2)	1006 (62.3)	1953 (73.9)	1165 (75.6)	1036 (79.1)	2201 (77.2)	936 (58.7)	1016 (59.1)	1952 (58.9)
Post-intervention	945 (53.3)	970 (49.2)	991 (50.6)	2906 (51.0)	971 (78.6)	944 (52.1)	1915 (65.5)	1103 (61.6)	994 (72.8)	2097 (66.9)	919 (51.8)	984 (54.9)	1903 (53.4)
Maternal age, y													
Pre-intervention	25.6 ± 6.1 (n = 505)	27.5 ± 5.4 (n = 536)	25.5 ± 6.1 (n = 527)	26.2 ± 6.0 (n = 1568)	25.9 ± 6.1 (n = 816)	27.1 ± 6.3 (n = 627)	26.4 ± 6.2 (n = 1443)	28.6 ± 5.8 (n = 881)	30.5 ± 6.3 (n = 819)	29.5 ± 6.1 (n = 1700)	30.1 ± 5.5 (n = 549)	29.5 ± 5.5 (n = 600)	29.8 ± 5.5 (n = 1149)
Post-intervention	25.6 ± 6.0 (n = 504)	27.8 ± 5.3 (n = 477)	25.1 ± 5.6 (n = 501)	26.1 ± 5.7 (n = 1482)	27.1 ± 6.6 (n = 760)	27.7 ± 6.3 (n = 476)	27.3 ± 6.5 (n = 1236)	28.6 ± 5.6 (n = 679)	29.9 ± 6.1 (n = 720)	29.3 ± 5.9 (n = 1399)	30.2 ± 5.9 (n = 476)	28.9 ± 5.8 (n = 539)	29.5 ± 5.9 (n = 1015)
Gestational age at birth, wk													
Pre-intervention	38.9 ± 1.3 (n = 505)	38.9 ± 1.2 (n = 536)	39.1 ± 1.3 (n = 527)	38.9 ± 1.3 (n = 1568)	38.5 ± 1.0 (n = 816)	38.4 ± 1.0 (n = 627)	38.4 ± 1.0 (n = 1443)	38.9 ± 1.0 (n = 881)	39.0 ± 1.2 (n = 819)	39.0 ± 1.1 (n = 1700)	39.3 ± 1.3 (n = 549)	39.4 ± 1.3 (n = 600)	39.3 ± 1.3 (n = 1149)
Post-intervention	38.8 ± 1.2 (n = 504)	38.6 ± 1.0 (n = 477)	38.8 ± 1.2 (n = 501)	38.8 ± 1.2 (n = 1482)	39.1 ± 1.0 (n = 763)	38.5 ± 1.0 (n = 492)	38.8 ± 1.0 (n = 1255)	38.7 ± 1.0 (n = 679)	39.0 ± 1.1 (n = 724)	38.8 ± 1.1 (n = 1403)	39.2 ± 1.2 (n = 476)	38.6 ± 1.0 (n = 540)	38.9 ± 1.1 (n = 1016)
Nulliparous													
Pre-intervention	504 (62.9)	536 (49.1)	525 (58.9)	1565 (56.8)	804 (57.7)	626 (46.5)	1430 (52.8)	880 (32.7)	817 (24.0)	1697 (28.5)	549 (40.6)	600 (46.3)	1149 (43.6)
Post-intervention	495 (61.0)	477 (52.0)	500 (57.0)	1472 (56.7)	748 (62.7)	474 (44.5)	1222 (55.6)	678 (27.9)	724 (29.1)	1402 (28.5)	476 (47.1)	540 (50.6)	1016 (48.9)
Total birth													
Pre-intervention	935 (54.5)	961 (55.9)	968 (54.5)	2864 (55.0)	947 (86.2)	1015 (62.2)	1962 (73.8)	1181 (74.9)	1045 (78.9)	2226 (76.8)	945 (58.5)	1031 (58.9)	1976 (58.7)
Post-intervention	957 (52.9)	983 (48.5)	994 (50.4)	2934 (50.6)	975 (78.4)	946 (52.0)	1921 (65.4)	1115 (61.2)	1004 (72.3)	2119 (66.4)	930 (51.2)	994 (54.5)	1924 (52.9)
Birth weight, g													
Pre-intervention	3057 ± 410 (n = 510)	3125 ± 424 (n = 537)	3063 ± 403 (n = 527)	3082 ± 413 (n = 1574)	2894 ± 777 (n = 814)	2919 ± 403 (n = 631)	2905 ± 641 (n = 1445)	3121 ± 422 (n = 885)	3152 ± 465 (n = 824)	3136 ± 443 (n = 1709)	3038 ± 433 (n = 553)	2962 ± 440 (n = 607)	2998 ± 438 (1160)
Post-intervention	3104 ± 399 (n = 506)	3133 ± 380 (n = 477)	3070 ± 388 (n = 501)	3102 ± 390 (n = 1484)	2914 ± 433 (n = 764)	2886 ± 402 (n = 492)	2903 ± 421 (n = 1256)	3090 ± 417 (n = 682)	3134 ± 427 (n = 726)	3113 ± 422 (n = 1408)	3035 ± 421 (n = 476)	2956 ± 433 (n = 542)	2993 ± 429 (n = 1018)

a Values given as number (percentage) or mean ± SD.

b Full-term normal birth was defined as normal vaginal delivery and gestational age more than 37 weeks.

**Table 2:** Surgical episiotomy rate pre- and post-intervention by center expressed as a percentage of full-term normal births.

Center	Births pre-intervention^a^	Births post-intervention^a^	Episiotomy pre-intervention, %	Episiotomy post-intervention, %	Risk difference (95% confidence interval) b	*P* value c
Thailand (all)	1568	1482	91.8	84.1	–7.7(–10.0 to –5.4)	< 0.01
Center 1	505	504	95.8	82.9	–12.9(–16.6 to –9.2)	< 0.01
Center 2	536	477	92.9	97.3	4.4 (1.8–7.0)	< 0.01
Center 3	527	501	86.7	72.9	–14.1(–18.9 to –9.2)	< 0.01
Philippines (all)	1274	1237	63.7	62.2	–1.5(–5.3 to 2.3)	0.44
Center 1	777	763	63.6	55.8	–7.8(–12.6 to –2.9)	< 0.01
Center 2	497	474	64	72.6	8.6 (2.8–14.4)	< 0.01
Malaysia (all)	1700	1403	46	39.3	–6.6(–10.1 to –3.2)	< 0.01
Center 1	881	679	60.6	51.1	–9.5(–14.5 to –4.6)	< 0.01
Center 2	819	724	30.3	28.3	–2.0(–6.5 to 2.6)	0.40
Indonesia (all)	1146	1016	53.5	51.2	–2.3(–6.5 to 1.9)	0.28
Center 1	546	476	45.2	38.4	–6.8(–12.8 to –0.8)	0.03
Center 2	600	540	61	62.4	1.4(–4.2 to 7.1)	0.63
Total sample	5688	5138	64.1	60.1	–4.0(–5.8 to –2.2)	< 0.01

a Full-term normal birth.

b Figures may not add up owing to rounding.

c Proportion *Z* test for 2 independent samples.

**Table 3:** Rate of intact perineum for full-term normal births for all women and nulliparous women.

Center	Nulliparous				Total sample				
	Pre-intervention, %	Post-intervention, %	RD (95% CI)	*P* value a	Pre-intervention, %	Post-intervention, %	RD (95% CI)	*P* value (unadjusted) a	Adjusted RD (95% CI) b
Thailand (all)							2.0 (0.8–3.3)	< 0.01	1.3 (–0.8 to 3.3)
Center 1	0.6	2.6	2.0 (0.01–4.0)	< 0.05	1.6	4.4	2.8 (0.7–4.9)	< 0.01	
Center 2	0.8	0.4	–0.4 (–1.7 to 1.0)	0.60	0.6	0.2	–0.4 (–1.1 to 0.4)	0.38	
Center 3	2.3	2.5	0.2 (–2.3 to 2.6)	0.88	4.2	7.6	3.4 (0.5–6.3)	0.02	
Philippines (all)							12.5 (8.9–16.1)	< 0.01	14.8 (2.5–27.1)
Center 1	4.9	30.8	26.0 (21.0–30.9)	< 0.01	29.6	38.9	9.4 (4.4–14.3)	< 0.01	
Center 2	0.0	9.2	9.2 (5.2–13.3)	< 0.01	6.1	23.5	17.3 (12.8–21.8)	< 0.01	
Malaysia (all)							–1.1 (–3.8 to 1.6)	0.43	0.5 (–2.8 to 3.8)
Center 1	0.0	1.1	1.1 (–0.4 to 2.5)	0.08	9.3	11.8	2.5 (–0.6 to 5.6)	0.11	
Center 2	1.5	5.7	4.2 (0.6–7.7)	0.03	28.2	22.5	–5.7(–10.0 to –1.4)	0.01	
Indonesia (all)							0.8 (–1.8 to 3.4)	0.54	1.4 (–1.9 to 4.6)
Center 1	1.8	3.6	1.8 (–1.2 to 4.8)	0.25	11.7	13.4	1.7 (–2.4 to 5.8)	0.41	
Center 2	2.2	1.1	–1.1 (–3.2 to 1.0)	0.32	8.1	8.1	0.04 (–3.1 to 3.2)	0.98	
Total	1.7	8.0	6.3 (5.0–7.5)	< 0.01	12.4	15.6	3.2 (1.9–4.6)	< 0.01	

Abbreviations: CI, confidence interval; RD, risk difference.

a Proportion *Z* test for 2 independent samples.

b Adjusted RD for whole-country outcomes only (adjusted for parity and maternal age).

**Table 4:** Rate of severe trauma pre- and post-intervention for full-term normal births in all women, nulliparous women, and those with and without episiotomy.^a^

Item	Pre-intervention	Post-intervention	RD (95% CI)	*P* value b
With and without episiotomy	213 (3.9)	96 (1.9)	–2.0(–2.7 to –1.4)	< 0.01
All women	n = 5433	n = 5034		
With and without episiotomy	163 (6.7)	71 (3.0)	–3.7(–4.9 to –2.5)	< 0.01
Nulliparous	n = 2418	n = 2337		
Without episiotomy	27 (1.4)	5 (0.2)	–1.1(–1.7 to –0.6)	< 0.01
All women	n = 1972	n = 2043		
With episiotomy	186 (5.4)	91 (3.0)	–2.3(–3.3 to –1.4)	< 0.01
All women	n = 3461	n = 2991		

a Values given as number (percentage) unless otherwise indicated.

b Proportion *Z* test for 2 independent samples.

**Table 5:** Suture material and technique used for skin closure at all centers for full-term normal births.^a^

Item	Pre-intervention (n = 5070)	Post-intervention (n = 4358)
Polyglycolic acid	60 (1.2)	429 (9.8)
Continuous suture	3624 (72)	2775 (64.0)
Continuous suture using polyglycolic acid	57 (1.1)	208 (4.8)

a Values given as number (percentage) unless otherwise indicated.
